# Review of the Environmental Impact of Digital Health: a Call for Structural Mitigation Measures

**DOI:** 10.1055/s-0046-1816613

**Published:** 2026-11-03

**Authors:** Parveen Kumar, Isabelle de Zegher

**Affiliations:** 1 Wageningen Environmental Research, Wageningen University & Researchthe Netherlands; 2 The Department of Advanced Computing Sciences, Maastricht UniversityMaastrichtthe Netherlands; 3 b!lobaTervurenBelgium

**Keywords:** Digital Health, Digital Health Technologies, Climate Change, Data Management Practices, Environmental Sustainability, Policy Interventions

## Abstract

**Objectives:**

Digital health technologies, including electronic health data, telemedicine, and artificial intelligence, have transformed healthcare delivery by improving accessibility, efficiency, and adaptability to climate risks. However, these innovations come with significant environmental costs, particularly greenhouse gas emissions associated with energy-intensive data storage, processing, and transmission. This scoping study aims to assess the dual impact of digital health: its potential to reduce digital healthcare related environmental footprints while also contributing to environmental degradation. Additionally, it evaluates existing and emerging policies addressing sustainability in the digital health sector.

**Methods:**

A scoping review was conducted to analyze scientific and grey literature, policy documents, related to digital health and environmental sustainability.

**Results:**

Key findings show that, while digital health enhances healthcare adaptability related to climate risks, the size of its impact on environmental resources is uncertain though sufficient to mandate actions. Voluntary mitigation measures are emerging in health-related software development, data lifecycle management, and hardware deployment. However, the review underlines a critical gap in digital health related regulations where environmental considerations and related mitigation measures remain largely unaddressed.

**Conclusions:**

While digital health offers promising solutions for climate-resilient healthcare, digital health environmental sustainability remains inadequately addressed. This study highlights the urgent need for policy interventions to balance digital health advancements with global climate mitigation goals. Policymakers, healthcare providers, and technology developers must prioritize green and sustainable practices, ensuring that digital health contributes to both healthcare innovation and environmental responsibility.

## 1. Introduction

Digital health technologies (DHT), encompassing telemedicine, artificial intelligence, wearable health devices, and electronic health records, have emerged as transformative tools in healthcare [ 1 – 5 ]the editors of the Clinical Information\nSystems (CIS. These technologies enable real-time data collection, analysis, decision-making and care management. As healthcare systems increasingly integrate these innovations, digital health has become a cornerstone of efforts to improve care delivery and public health outcomes [ 6 – 8 ]. However, the widespread adoption of these technologies is not without environmental consequences. For instance, the infrastructure supporting digital health, such as data centers, relies heavily on energy-intensive computing and storage systems [ 9 ]. Furthermore, the production and disposal of digital health devices contribute to e-waste, natural resource depletion, soil and water contamination and greenhouse gas (GHG) emissions [ 10 – 12 ]a growing literature is shedding light on the highly polluting nature of the digital industry and how it exacerbates health inequalities. Thus, the environmental footprint of DHTs should be considered when assessing their overall value to healthcare systems. The objectives of this article are to: (1. As healthcare systems worldwide strive to achieve higher quality of care with ‘Net Zero’ impact on climate, the environmental footprint of digital health is an emerging area of attention. This intersection of digital transformation and environmental sustainability calls for a closer investigation to align digital health innovations with climate mitigation goals.

Digital health holds potential for reducing healthcare’s environmental impact, particularly through telemedicine, digital monitoring, and access to quality healthcare [ 13 , 14 ]. Telemedicine, which enables remote consultations, minimizes the need for patient travel and reduces GHG emissions associated with transportation. For example, a 2023 study by Rodler *et al.* [ 15 ], demonstrated the significant environmental benefits of telemedicine, reporting approximately 68,465,481 consultations that resulted in savings of 691,825 tons of CO₂ emissions and a reduction of over 3.31billion kilometers in travel distance. Similarly, wearable devices and mobile health applications support continuous patient monitoring, decreasing the frequency of resource-intensive hospital visits. These advancements collectively contribute to a lower carbon footprint in healthcare delivery. However, despite these benefits, DHTs also present notable environmental challenges. Data centers, which store and process the vast quantities of generated health data, are significant energy consumers, often relying on fossil fuel-based energy sources [ 9 ]. The International Energy Agency’s Electricity 2024 report highlights that the global data center industry is estimated to account for approximately 1% of global electricity consumption [ 16 ]. Moreover, the computational demands of artificial intelligence (AI) training and model deployment can be substantial. Studies have shown that training a single AI model can emit as much CO₂ as five cars over their entire lifetimes [ 17 ]. As the healthcare sector increasingly integrates digital technologies, addressing these environmental downsides is critical to ensuring that the benefits of digital health are achieved sustainably [ 18 ].

A critical yet often overlooked aspect of the environmental impact of digital health is the rebound effect. As digital health services scale, the increased energy consumption from expanded use can partially or entirely offset the benefits of emissions reductions [ 19 ]. For example, while enhanced digital healthcare accessibility improves service delivery, it may also lead to the overuse of services, thereby increasing data transmission and processing demands. Furthermore, the frequent updates and replacements of digital health devices can exacerbate resource consumption and contribute to increased electronic waste generation [ 10 ]a growing literature is shedding light on the highly polluting nature of the digital industry and how it exacerbates health inequalities. Thus, the environmental footprint of DHTs should be considered when assessing their overall value to healthcare systems. The objectives of this article are to: (1.

As highlighted earlier, the intersection of digital transformation and environmental sustainability calls for closer examination to ensure that digital health objectives align with climate mitigation goals. Addressing this critical intersection, this study investigates the environmental impacts of digital health, focusing on balancing their positive contributions with their negative consequences. Specifically, this study examines:

(i) the environmental benefits of digital health, such as telemedicine and digital monitoring, in reducing emissions and resource-intensive activities,
(ii) the environmental costs, including energy demands and resource-intensive processes associated with data storage, processing, and device production, and
(iii) policy and governance gaps that defer the integration of sustainability into digital health practices limiting the health sector’s ability to contribute to climate mitigation goals effectively.

Several studies demonstrate the benefits of DHTs in supporting emergency healthcare delivery, particularly in response to climate hazards such as warming, floods, droughts, and tropical cyclones, which are projected to increase in frequency and intensity [ 20 , 21 ]with consequences for human food security and mental health status. Ubiquitous digital devices, such as smartphones, have the potential to manage existing and emerging climate-related crises, given their ability to enable rapid response, instant communication, and knowledge sharing.\nObjective: This scoping review aimed to identify digital apps being used to capture or address climate change impacts on food security and mental health to inform the development of a digital citizen science initiative.\nMethods: A scoping review was conducted using 3 peer-reviewed databases (PubMed, IEEE Xplore, and Web of Science. The efficacy of DHTs to manage climate influenced public health crises is a subject that alone requires a comprehensive review and thus is not covered in this study. Instead, we focus on the environmental costs and benefits of DHTs applications for primary and continued health care services.

By addressing the intersection of digital transformation and environmental sustainability, this review study provides actionable insights for policymakers, healthcare providers, and technology developers. It emphasizes the critical need to integrate environmental sustainability into digital health advancements, ensuring alignment with global climate change mitigation goals. The review is organized as follows: the methodology section outlines the literature search and analysis process, followed by an exploration of the results. The discussion section interprets these findings, offering recommendations for structural sustainability practices. The review concludes with a call to action, urging stakeholders across sectors to collaboratively advance sustainable digital health practices.

## 2. Methods

### 2.1 Literature Search

Between October and December 2024, a systematic search was conducted across Web of Science, PubMed, Scopus, and Google Scholar to identify relevant literature, including both peer-reviewed and gray literature. Keywords, detailed in Table 1 , were designed to capture a comprehensive range of studies addressing themes such as “digital health and environmental impact”, “health data management and energy use” and “AI and sustainability in digital health.”

**Table 1:** List of the keywords to search databases and Google search engine.

Keyword Group	Keywords
Digital Health	Digital health, e-health, telehealth, telemedicine, mobile health, virtual care, digital healthcare technology
Artificial Intelligence	Artificial intelligence, machine learning, deep learning, computational health, AI-driven healthcare, predictive models
Data Centers	Data center, server farm, cloud storage, server infrastructure, data storage, computing resources, energy-intensive data, data center carbon footprint
Health Data Management	Health data management, data lifecycle, data processing, data storage, data transmission, health record storage, big data in healthcare, electronic health records
Environmental Impact	Environmental impact, carbon footprint, CO2 emissions, greenhouse gas emissions, climate impact, energy consumption
Energy Consumption	Power usage, electricity consumption, energy efficiency, resource demand, energy usage, power-hungry technology, energy footprint
Sustainable Healthcare	Sustainable healthcare, environmental sustainability, renewable energy in health technology, green IT in healthcare, low-carbon health practices, responsible data management
Policy Interventions	Policy interventions, regulations on digital health, environmental policy in health technology, sustainable policies in healthcare, health regulations

A variety of search strategies were applied to ensure a thorough exploration of the topic. These strategies, including databases searched, key search strings, and the number of articles retrieved, are summarized in Table 2 . The scope of the search was expanded to include healthcare related literature, ensuring the inclusion of all pertinent studies. Exclusion criteria, outlined in Table 3 , were applied to refine the dataset further by filtering initially identified literature based on factors such as specificity of the topic, type of literature, language, publication year, and relevance to the research questions.

**Table 2:** Search strategy for literature review, including database searched, key strings used and number of relevant publications retrieved.

**Narrative**	**Database**	**Search String**	**Number before applying filters**	**Number eligible for final screening**
Digital Health and Environmental Impact	Web of Science	("digital health*" OR "e-health" OR "ehealth" OR "telehealth" OR "telemedicine" OR "mhealth" OR "virtual care" OR "digital healthcare technolog*") AND ("artificial intelligence" OR "AI" OR "machine learn*" OR "deep learn*" OR "comput?tional health" OR "AI-driv* healthcare" OR "predictive model*") AND ("environment* impact" OR "carbon footprint" OR "CO2 emission*" OR "greenhouse gas* emission*" OR "climate impact" OR "energy consum*") AND ("data cent*" OR "server farm*" OR "cloud storag*" OR "server infrastructur*" OR "data storag*" OR "comput* resourc*" OR "energy-intensive data") AND ("sustainable healthcare" OR "environment* sustainab*" OR "renewable energy in health* technolog*" OR "green IT in healthcare" OR "low-carbon health* practice*" OR "responsible data manag*")	8	3
	PubMed	11	6
	Scopus	798	18
Health Data Management and Energy Use	Web of Science	("health data manag*" OR "data lifecycle" OR "data process*" OR "data storag*" OR "data transmiss*" OR "health record* storag*" OR "big data in healthcare" OR "electronic health record*") AND ("energy consum*" OR "power usag*" OR "electricity consum*" OR "resource demand*" OR "power-hungry technolog*" OR "energy-intensive data") AND ("carbon footprint" OR "greenhouse gas* emission*" OR "environment* impact")	43	5
	PubMed	2	1
	Scopus	1766	17
AI in Healthcare and Environmental Sustainability	Web of Science	("artificial intelligence" OR "AI" OR "machine learn*" OR "deep learn*" OR "comput?tional health" OR "AI-driv* healthcare") AND ("energy consum*" OR "carbon footprint" OR "resource demand*" OR "ener* usag*") AND ("sustainab* healthcare" OR "green technolog*" OR "environment* sustainab*" OR "renewable energy" OR "responsible data manag*")	583	4
	PubMed	18	2
	Scopus	44281	4

**Table 3:** Exclusion Criteria for Literature Searches.

**Criterion**	**Description**
Irrelevant focus	Studies focusing solely on health outcomes of digital health without reference to environmental impacts.
Technological discussion without sustainability context	Studies discussing digital health technology in terms of functionality, adoption, or user experience without links to sustainability or environmental impact.
Non-peer-reviewed content	Blog posts, news articles, or other non-peer-reviewed content lacking scientific rigor (excluding official reports or white papers with valuable data).
General health and sustainability topics	Documents discussing health and sustainability without specific reference to digital health technologies.
Lack of environmental impact analysis	Studies emphasizing the benefits of digital health without evaluating their environmental implications.

The inclusion criteria consisted of diverse document types, such as book chapters, preprints, conference proceedings, peer-reviewed journal articles and policy documents (e.g., regulations, directives, strategy papers, etc.). The initial screening of the peer-reviewed and grey literature was conducted by the first author, who identified studies meeting the inclusion criteria and prepared a preliminary data base for literature review. To reduce bias and ensure thorough coverage, the second author subsequently reviewed the preliminary data base and findings of the first author by re-evaluating the included materials and findings. The reference lists of all included articles were also systematically reviewed by both authors in sequence to identify additional relevant materials. This step-by-step process allowed for in-depth cross-validation, ensuring that key studies were not overlooked and that the findings were robust and reliable.

After removing duplicates, the remaining documents were screened for relevance, resulting in the inclusion of 43 peer-reviewed publications and 28 policy documents, as detailed in Appendix I and II. These selected publications were later analyzed for their content and deductively coded into predefined categories, including: title, publication year, type of publication (e.g., book chapter, journal article), publication venue (e.g., journal name), author(s), abstract, keywords, Etc. This structured approach ensured the development of a robust dataset, enabling an in-depth examination of the environmental implications of DHTs.

### 2.2 Selection of Policies, Initiatives, and Strategy Papers related to Digitization and Digital Health

To complement the systematic peer-reviewed literature review described above, grey literature was searched using Google with the keywords outlined in Table 1 to identify initiatives and policies relevant to the research question: Which policies or initiatives address climate mitigation efforts associated with digital health? The review includes policies from nations in both the Global North (e.g., European countries, UK, US, Japan) and the Global South (e.g., India, Brazil) to ensure a balanced and comprehensive analysis. Appendix II provides a comprehensive list of selected policies, initiatives, and strategy papers related to digitization and digital health that were reviewed as part of this study.

While the search includes documents in many formats with different intended applications, priority was given to directives, policies, and strategic papers addressing digital aspects that may impact the health sector and explicitly focus on sustainability and climate mitigation in digitalization. For the grey literature search, a combination of keyword strategies was applied, and all returned links were examined, with a detailed review of at least the first few web pages of search results. Exclusion criteria were applied to filter out directives, laws, policies, initiatives, and frameworks that did not explicitly address the intersection of digitization, digital health and climate mitigation measures. For each potential policy or initiative, the following information was collected: institutional origins, policy type, focus areas, objectives, etc. All retrieved links and documents were systematically reviewed to identify content relevant to the research question. This approach ensured the inclusion of high-quality and contextually significant resources, facilitating a nuanced understanding of how digital health policies intersect with climate mitigation efforts.

### 2.3 Review and Content Analysis

The selected articles and policies underwent a methodological review to ensure their consistency, depth, and relevance to the research objectives through the following steps:

- Abstracts, summaries, and keywords of the documents were carefully examined to confirm alignment with the research scope. Only those meeting the inclusion criteria were shortlisted for further analysis;
- Each shortlisted document was meticulously reviewed to extract detailed insights. The review focused on predefined themes, including environmental impacts, GHG emissions, policy implications, and sustainability measures in digital health. This thematic clustering enabled an organized synthesis of key findings;
- To ensure the reliability and relevance of insights and to minimize bias, the coding process was developed based on prior steps, allowing two independent reviewers to validate the results. Any discrepancies were collaboratively discussed and resolved, maintaining the integrity of the review process.

Insights from the review were consolidated into broader categories that facilitated a comprehensive understanding of the results including actionable insights and trends. Key components included categorization of environmental impacts, where papers were classified based on their discussion of energy use, GHG emissions, and mitigation strategies in the digital health sector. Policies were assessed for their relevance to digital health sustainability, climate mitigation measures, and alignment with broader environmental goals. Additionally, effective strategies and frameworks for reducing the environmental footprint of digital health were identified and highlighted.

## 3. Results

### 3.1 Overall Trends in Literature

This study analyzed 43 peer-reviewed publications to examine the environmental impacts of DHTs, highlighting a growing interest in the intersection of digital health and climate change in recent years. Particularly, 62% of the reviewed studies were published after 2022, indicating increasing recognition of the sustainability challenges associated with digital health.

### 3.2 Geographical Trends in Digital Health and Climate Change

The geographical distribution of reviewed publications on digital health and climate change highlights a notable gap in the literature, with a predominant focus on Europe and North America (predominantly USA), with additional contributions from Australia, Asia and few studies from Africa. Many studies adopt a global or multi-regional perspective. European research dominates, with 12 studies from the UK, alongside notable contributions from Germany, Netherlands, Poland, France, Italy and Austria, and a total of nine studies from the broader EU region. These studies often address topics like digital health sustainability, teleconsultations, and environmental impacts. North America, represented by five studies from the USA and 1 from Canada, primarily focuses on healthcare, radiotherapy carbon footprints, and system-specific sustainability initiatives. In Asia, the representation includes three studies from China, two studies from Japan, and one study from India, highlighting regional efforts in telehealth and AI integration. Additionally, the Middle East contributes with one study from the UAE, one from Saudi Arabia. Africa and South America add one study each that investigates DHT. Africa remains underrepresented. In contrast, Australia contributes four studies, emphasizing the environmental and healthcare impacts within its unique context. While Europe and North America dominate the research landscape for this topic, the limited representation from Africa, Asia, and the Global South highlights a significant gap. This underscores the need for further studies in these underrepresented regions to ensure a more comprehensive understanding of digital health’s global environmental and societal impacts.

### 3.3 Environmental Impacts identified in Digital Health Literature

This review shows that the environmental impacts associated with DHT are diverse. Energy consumption is a significant concern, driven by the demand from data centers, computing hardware, and network infrastructure. Several studies [ 12 , 18 , 22 , 23 ] emphasize the adoption of energy-efficient hardware, the optimization of data storage, and the development of energy-conscious software as critical strategies to mitigate these impacts. Similarly, the reduction of carbon emissions emerges as a major focus, with research advocating for the development of low-carbon software (AI and non-AI) and comprehensive assessments of the carbon footprint of digital health solutions to align with global decarbonization goals. The growing challenge of e-waste presents a significant environmental concern, particularly as the adoption of DHTs continues to expand. Discarded electronic health devices and digital infrastructure contribute to hazardous waste accumulation, posing risks such as toxic chemical leakage, soil and water contamination, and long-term ecological harm. Without effective management strategies, the increasing reliance on DHTs could further exacerbate resource depletion and pollution, particularly in regions lacking robust recycling infrastructure. To mitigate these risks, it is essential to extend the lifespan of electronic equipment, develop efficient recycling and refurbishment programs, and implement sustainable disposal practices. Encouraging a circular economy approach, which emphasizes reuse, responsible material sourcing, and closed-loop production cycles, can significantly reduce environmental impact.

### 3.4 Key Themes in the Digital Health and Climate Change Nexus

Thematic analysis of the selected literature showed several repeating themes in the digital health domain, highlighting both the opportunities and challenges presented by DHTs in addressing climate related issues.

One noticeable theme is the positive impact of telehealth and medical devices as a key strategy for reducing carbon emissions by minimizing patient travel and in-person visits, and enabling remote monitoring. Numerous studies [ 14 , 24 , 25 ] emphasize their significant role in improving healthcare accessibility and preserving patient outcomes while simultaneously reducing environmental footprints.

Another theme relates to Electronic health records (EHRs) systems that reduce paper consumption while improving communication between healthcare staff [ 26 ]. The most noticeable trend, however, in software solutions is the application of artificial intelligence (AI). AI and more specifically generative AI with Large Language Models (LLMs) play an increasing role in healthcare delivery [ 13 ]. These technologies are applied in various contexts, including prevention, diagnosis and therapeutics enabling optimization of healthcare workflows.

Despite these benefits, the analysis also highlights negative environmental impacts of DHTs. The amount of digital space required to store personal health records including genetics data and medical imaging and the ever-growing quantity of data and computational resources needed to train and execute AI models mandate the growing capacity of data centers. Most AI models in the digital health domain rely on data centers, which consume substantial energy for cooling and operation, thus contributing to greenhouse gas emissions. As a result, the digital health sector is increasingly recognized as a polluting and energy-intensive industry.

Voluntary mitigation measures are gaining traction in areas such as software optimization (e.g., software carbon-tracker, designing low-energy AI algorithms), hardware deployment (e.g., modular and energy-efficient devices), and sustainable data management (e.g., reduce storage of unused data). However, these practices remain fragmented and require stronger regulatory frameworks to achieve widespread adoption.

A critical gap identified in the review is the absence of policies and mitigation measures addressing the environmental impacts of DHTs. Only 15% of the reviewed academic studies explicitly discussed policy interventions for sustainable digital health practices, highlighting a significant governance deficit, further confirmed by the analysis described in the next section.

#### Analysis of policies, regulations, and guidelines

A comprehensive analysis of 38 policy documents, strategies, frameworks, and directives was conducted. These documents included contributions from regions such as the United States (n=4), Canada (n=2), Australia(n=2), Japan(n=2), South Korea(n=2), India(n=2), China(n=2), the United Kingdom (n=7), and the European Union(n=15). Of these, only 28 were viewed as relevant to the intersection of digital health and climate change. Most of them (n=28 including 12 regulations and 16 strategy papers/guidelines) address climate change and mitigation measures generally. Some (n=17) mentioned digital technology including data centers, AI, Internet of Things (IoT) without specific reference to digital health. Specifically, the EU Artificial Intelligence Act, the most recent regulation, emphasizes the development of energy efficient AI systems, addressing the energy-intensive nature of machine learning processes. When mentioned, health was referred to only in the context of the impact of climate change on public health.

A minority of policy documents (i.e., n=8, including three regulations, five strategy papers/guidelines) specifically relate to digital health, but none mention impact on climate or emissions mitigation measures. Notably the UK’s “Delivering a ‘Net Zero’ National Health Service,” mentions the benefit of digitalization for low-carbon transformation without considering the negative impact of data centers. Except for the AI Act, the EU regulations related to digital aspects, including the Digital Market Act (DMA), the Digital Services Act (DSA), the Data Governance Act (DGA) and the Data Act (DA), do not mention impact from data centers neither discuss any climate related mitigation measures. This is also true for the European Health Data Space regulation (EHDS) and the WHO Global strategy on digital health [ 22 ]. The analysis of selected policies and regulations reveals that the fragmented nature of current policy frameworks underscores the urgent need for comprehensive policies that align digital health and climate change objectives. This alignment is essential to support sustainable practices within DHTs and support broader environmental and healthcare goals.

## 4. Discussion

Digital health technologies (DHTs) have emerged as transformative tools in modern healthcare, enhancing efficiency, accessibility, and outcomes. However, these technologies also pose noteworthy environmental challenges. This dual role emphasizes the importance of critically examining their benefits and limitations to foster sustainable practices that align healthcare advancements with global climate mitigation efforts.

Digital health provides substantial benefits across various dimensions of healthcare. EHR systems, for instance, have significantly reduced reliance on paper-based systems, streamlining patient management and improving communication among healthcare staff [ 26 ]. Their adoption reduces material waste while facilitating more efficient clinical workflows. Telemedicine has represented a pivotal structural change in healthcare particularly in addressing health care access disparities. By enabling remote consultations, telemedicine ensures continuity of care in geographically isolated regions, reducing the need for patient travel, which may be costly or physically difficult for some patients. Studies have demonstrated the potential to cut significant amounts of CO₂ emissions by reducing travel-associated carbon footprints, while maintaining and improving the quality of care [ 14 , 15 , 25 , 27 , 28 ]. The Internet of Things (IoT) has further transformed healthcare by enabling real-time monitoring and improving energy efficiency within healthcare facilities, ensuring that critical resources are available where and when they are needed. Application of AI has a growing role in healthcare delivery [ 13 ] and in supporting adaptive healthcare systems in the face of climate challenges [ 29 , 30 ]. For instance, AI enables predictive analytics which, first and foremost improves patient outcomes, but also optimizes resource allocation and reduces waste.

While the benefits of digital health are well-documented, their environmental costs cannot be underestimated. More specifically, telemedicine has the lowest net footprint, thanks to travel savings far exceeding its digital overhead [ 31 ], while AI models development produce relatively high amounts of emissions due to computing power needed for model training [ 32 ]. Data centers, which serve as the backbone of digital health infrastructure contribute to emissions and energy consumption. However, their energy demands are being amplified by AI technologies, particularly Deep Learning (DL) models and emerging Large Language Models LLM, which require substantial computational resources during training and inference phases [ 33 ].

Several studies provide an overview of the evolution of GHG emission estimates for data centers across sectors, illustrating both progress in emission reductions and persistent challenges and uncertainties. A study by Belkhir & Elmeligi in 2018 [ 34 ] projected that the ICT sector’s share of global GHG emissions would rise from 1.4–1.9% in 2010 to 3–3.6% in 2020, surpassing emissions from air transport. They estimated that data centers contributed 33% of the ICT sector’s carbon footprint in 2010, increasing to 45% in 2020, with absolute emissions nearly 500 Mt-CO2eq. Unchecked growth could push ICT emissions beyond 14% of global GHG emissions by 2040. However, recent studies vary, with a 2024 analysis of German data centers predicting a 13% emission rise from 2020 to 2030 and Scope 3 emissions increasing from 17% to 30% [ 35 ] the European Union aims for climate neutral data centers by 2030. To map potential developments of emissions associated with data centers to the year 2030, we develop a generic data center greenhouse gas (GHG. A more recent study estimates that data centers contribute less than 1% of global energy-related GHG emissions, indicating a lower impact than earlier projections [ 36 ]. However, many uncertainties remain, as demonstrated by the Guardian’s analysis [ 37 ] showing that GHGs emissions from data centers are 662% or 7.62 times higher than officially reported, as they use renewable energy credits. In addition, GHG emission are expressed as a percentage of total energy related emissions, which depend on emission trajectories of all other sectors. Ultimately, the rate of adoption of renewable energy by the data centers as well as the impact of expansion of data volume and AI on computing power on data centers are difficult to predict.

The healthcare sector generates 30% of the world’s data volume within data centers, i.e., between 0.3% to 1% of the total global GHG emissions. That share of data volume is projected to rise to 36% by 2025 [ 28 ] and can be expected to increasingly adopt data and computationally greedy AI solutions. This increased demand will increase the proliferation of data centers and underscores the need to address the environmental implications of DHTs. To preserve their benefits while minimizing their impact on climate change, it is critical to prioritize environmental responsibility [ 12 ] and to implement mitigation strategies [ 23 , 38 ] as well as digital sobriety measures contributing to the ambitious goal of Net Zero Healthcare [ 39 ].

Mitigation measures to address GHGs emissions were proposed by the American Meteorological Society (AMS) [ 40 ] and later reiterated in their 2020 I-CLIMATE framework [ 18 ]. These measures include conservation and circular economy practices; research, development, and deployment of new technologies that track and minimize GHGs emissions, enabling environmentally friendly decision-making by health staff and encouraging choices that lower emissions; and regulations supporting green practices. Additionally, AMS proposed two broader measures: raising public awareness and introducing pricing mechanisms for GHG emissions to create incentives for emission reductions, that are not further discussed in this paper.

Our review shows that voluntary measures emerging within the digital health domain primarily focus on three areas: adopting sustainability practices toward a circular economy, implementing new green technologies, and promoting green choices in data management. The three key areas should complement non-voluntary measures such as mandating renewable energy adoption in data centers and charging companies for GHG emissions.

Circular economy practices in the digital health sector: Extending the lifespan of medical devices, enhancing recycling efforts, and minimizing material and energy consumption are critical strategies. Health-specific interventions, such as optimizing the use of radiopharmaceuticals and reducing unnecessary imaging procedures, contribute to resource efficiency without compromising patient outcomes[ 30 , 41 – 43 ].

Create an ecosystem of green technologies: Carbon-tracking tools, such as those offered by Amazon and Google, enable stakeholders to evaluate and reduce emissions associated with DHT. Tools like Green Algorithm for High-Performance Computing (GA4HPC) further support energy-efficient operations. These technologies must be complemented by policy frameworks that incentivize their adoption [ 12 , 44 ]methods, and tools to evaluate the carbon footprint of digital health interventions for environmentally sustainable healthcare. MATERIALS AND METHODS: Medline (Ovid.

Responsible data management practices: Addressing the “datafication” culture in healthcare, where excessive data generation leads to underutilization, is essential [ 45 ]. Sustainable data management practices focus on lifecycle optimization, interoperability, and minimizing data storage requirements. The World Economic Forum [ 46 ] reports that 97% of healthcare data remains unused, highlighting the need for efficient data collection and reuse to reduce environmental waste. Source data must be retained for healthcare continuity and regulatory compliance. However, an estimated 25–30% of this data consists of duplicates, with up to 30% of electronic health record information being redundant [ 47 , 48 ]. Additionally, 10–20% of data is resulting from transformation of source data for analytics and AI training and could be deleted after use [ 49 ], with “just in time data curation” from interoperable health records and traceability from source data [ 50 ]. While awareness of the risks associated with “big data, big waste” [ 51 ], is increasing, sustainable data management ensuring data is stored, processed, and accessed with minimal environmental impact, resilience, and cost-effectiveness receives little attention in academic and political spheres [ 37 , 52 , 53 ].

These interventions must be supported by comprehensive policy frameworks and regulatory mechanisms to ensure scalability and effective implementation across the healthcare and digital health sectors. However, regulations explicitly addressing digital health’s role in climate mitigation or promoting environmentally conscious practices within healthcare remain notably absent and represent a considerable barrier to sustainable digital health.

This study demonstrates that regulations, policy papers, and guidelines addressing the resource consumption and environmental impact of digital technology generally do not consider the environmental impact of health and certainly digital health as a priority; they give limited attention to data centers, except for few regulations focusing on that specific topic. Most disturbing is the fact that even recent regulations and strategies across the globe related to digital health do not discuss environmental impact and potential mitigation measures to decrease this impact, while support of regulators are critically needed to ensure fast uptake of sustainable practices [ 23 ]. Existing regulations, such as the AI Act and EHDS in Europe, focus on digitalization but largely neglect environmental considerations. This regulatory gap hinders the adoption of mitigation strategies, delaying progress toward a sustainable healthcare system. Moreover, global collaborations should prioritize harmonizing digital health and climate policies to ensure scalability and rapid implementation.

DHTs are both an opportunity and a challenge in addressing climate change; their impact involves a variety of stakeholders, policies and guidelines, sometimes with divergent or even incompatible objectives and expectations [ 10 ]a growing literature is shedding light on the highly polluting nature of the digital industry and how it exacerbates health inequalities. Thus, the environmental footprint of DHTs should be considered when assessing their overall value to healthcare systems. The objectives of this article are to: (1. Mitigation measures described in this paper are needed to minimize the negative impact on GHG emissions and maximize the contribution to Net Zero Healthcare. Additional specification, research, piloting and deployment of such measures across the health sector are needed to refine these measures and assess their scalability across different healthcare systems. To ensure scalability and fast uptake of the measures, existing and emerging legislations related to AI and health data management should be modified to implement them. By aligning digital health innovations with environmental sustainability goals, the healthcare sector can become a leader in addressing the global climate crisis.

## 5. Conclusions

This study offers a comprehensive examination of the environmental impacts of digital health technologies and their contribution to climate change, alongside an evaluation of emerging sustainability practices from the digital health community. It highlights discrepancies between the green agenda of some regions and the lack of concrete action in the regulations to address these challenges. By bringing attention to both the issues and the potential of DHTs, the study aims to raise awareness among key stakeholders regarding the environmental footprint of digital health advancements.

The review calls for the urgent need for embedding sustainable practices into the lifecycle of digital health technologies and practices, from development to deployment. This call is particularly timely, as technologies are advancing and applications are expanding at unprecedented rates. Integration and implementation of sustainable practices throughout the development of the infrastructure of these technologies will be more effective, less difficult and less costly than any post hoc attempt to improve established infrastructure. The findings and suggestions are intended to guide policymakers, healthcare providers, and technology developers in adopting policy interventions and industry standards that prioritize not only energy efficiency, renewable energy integration, but also environmental responsibility of digital health initiatives. Furthermore, the study advocates for improved data management practices, emphasizing the importance of transitioning away from unsustainable data use and toward resilient, low-impact systems.

Ultimately, this review aligns digital health initiatives with global climate change mitigation goals, advocating for technological progress that harmonizes with environmental sustainability. By addressing these critical intersections, it provides a roadmap for achieving a greener, more responsible future for digital health.
